# Concordance between Three Homologous Recombination Deficiency (HRD) Assays in Patients with High-Grade Epithelial Ovarian Cancer

**DOI:** 10.3390/cancers15235525

**Published:** 2023-11-22

**Authors:** Elena Fountzilas, Kyriaki Papadopoulou, Thomas Chatzikonstantinou, Georgios Karakatsoulis, Pantelis Constantoulakis, Aikaterini Tsantikidi, Georgios Tsaousis, Sofia Karageorgopoulou, Anna Koumarianou, Davide Mauri, Anastasios Ntavatzikos, Zacharenia Saridaki, Georgios Petrakis, Florentia Fostira, George Fountzilas, Michalis Liontos

**Affiliations:** 1 Department of Medical Oncology, St. Lukes’s Hospital, 55236 Thessaloniki, Greece; 2 Medical Oncology, German Oncology Center, European University Cyprus, 1516 Nicosia, Cyprus; 3 Laboratory of Molecular Oncology, Hellenic Foundation for Cancer Research/Aristotle University of Thessaloniki, 54006 Thessaloniki, Greece; kyriakipapadopoulou@hotmail.com; 4 Institute of Applied Biosciences, Centre for Research and Technology Hellas, 57001 Thessaloniki, Greece; thomas.chatzikonstantinou@certh.gr (T.C.); g.karakatsoulis@certh.gr (G.K.); 5 Genotypos M.S.A., 11528 Athens, Greece; pconstantoulakis@genotypos.gr; 6 Genekor Medical S.A., 15344 Athens, Greece; tsantikidi.k@genekor.com (A.T.); gtsaousis@genekor.com (G.T.); 7 Third Department of Medical Oncology, IASO Clinic, 15123 Athens, Greece; skarageorg@hotmail.com; 8 Hematology Oncology Unit, Fourth Department of Internal Medicine, Attikon University Hospital, Medical School, National and Kapodistrian University of Athens, 12462 Athens, Greece; akoumari@yahoo.com (A.K.); dmaal2@yahoo.gr (A.N.); 9 Department of Medical Oncology, University Hospital of Ioannina, 45500 Ioannina, Greece; dvd.mauri@gmail.com; 10 1st Oncology Department, Metropolitan Hospital, 18547 Athens, Greece; zeniasar@gmail.com; 11 Oncology Department, “Asklepios DIAGNOSIS”, 71303 Heraklion, Greece; 12 Pathology Department, University General Hospital of Thessaloniki AHEPA, Medical School, Aristotle University of Thessaloniki, 54636 Thessaloniki, Greece; georgiospetrakismd@gmail.com; 13 InRASTES, National Centre for Scientific Research “Demokritos”, 15341 Athens, Greece; florentia_fostira@hotmail.com; 14 Aristotle University of Thessaloniki, 54124 Thessaloniki, Greece; fountzil@auth.gr; 15 Department of Medical Oncology, German Oncology Center, 4108 Limassol, Cyprus; 16 Department of Clinical Therapeutics, Alexandra Hospital, School of Medicine, National and Kapodistrian University of Athens, 11528 Athens, Greece; mliontos@gmail.com

**Keywords:** biomarker, concordance, epithelial ovarian cancer, homologous recombination deficiency, enomic instability

## Abstract

**Simple Summary:**

In patients with epithelial ovarian cancer, the gold standard Myriad MyChoice^®^ CDx assay is used to assess homologous recombination deficiency (HRD), as a biomarker predicting benefit from a targeted treatment, poly (ADP-ribose) polymerase inhibitors (PARPi). Having multiple available assays to identify patients with HRD-positive tumors is critical to ensure sufficient diagnostic capacity across different countries, and to provide greater variety for clinicians and diagnostic laboratories. Our aim was to evaluate the concordance between Myriad MyChoice and two alternative HRD assays (AmoyDx HRD Focus NGS Panel and OncoScan™) in patients with epithelial ovarian cancer. In the same tumor tissue samples that were previously assessed with the Myriad MyChoice^®^ CDx assay, the two alternative platforms evaluated HRD status in a blinded manner. Indeed, our study demonstrated high concordance between the Myriad MyChoice assay and each assay under evaluation, thus providing alternative options for HRD testing.

**Abstract:**

Our aim was to evaluate the concordance between the Myriad MyChoice and two alternative homologous recombination deficiency (HRD) assays (AmoyDx HRD Focus NGS Panel and OncoScan™) in patients with epithelial ovarian cancer (EOC). Tissue samples from 50 patients with newly diagnosed EOC and known Myriad MyChoice HRD status were included. DNA aliquots from tumor samples, previously evaluated with Myriad MyChoice and centrally reassessed, were distributed to laboratories to assess their HRD status using the two platforms, after being blinded for the Myriad MyChoice CDx HRD status. The primary endpoint was the concordance between Myriad MyChoice and each alternative assay. Tumor samples were evaluated with an AmoyDx^®^ HRD Focus Panel (*n* = 50) and with OncoScan™ (*n* = 43). Both platforms provided results for all tumors. Analysis showed that correlation was high for the Myriad MyChoice GI score and AmoyDx^®^ HRD Focus Panel (r = 0.79) or OncoScan™ (r = 0.87) (continuous variable). The overall percent agreement (OPA) between Myriad MyChoice GI status (categorical variable) and each alternative assay was 83.3% (68.6–93.3%) with AmoyDx and 77.5% (61.5–89.2%) with OncoScan™. The OPA in HRD status between Myriad MyChoice and AmoyDx was 88.6% (75.4–96.2). False-positive rates were 31.6% (6/19) for AmoyDx GI status and 31.9% (7/22) for OncoScan™, while false-negative rates were 0% (0/28, AmoyDx) and 11.1% (2/18, OncoScan™) compared with the Myriad MyChoice GI status. While substantial concordance between Myriad MyChoice and alternative assays was demonstrated, prospective validation of the analytical performance and clinical relevance of these assays is warranted.

## 1. Introduction

Ovarian cancer is the fifth most common cause of cancer mortality worldwide [1]. The majority of patients are diagnosed with advanced-stage ovarian cancer, resulting in poor prognosis. While a significant proportion of patients with advanced disease will respond to standard platinum-based chemotherapy, 70% of patients will eventually recur. Before recent advances in the treatment of ovarian cancer, the 5-year survival of stage III–IV ovarian cancer was less than 30% [2]. Recent advances in precision oncology, including the use of targeted agents, have changed the therapeutic landscape of patients with advanced ovarian cancer, while significantly improving their prognosis.

Poly (ADP-ribose) polymerase inhibitors (PARPi) have been associated with improved clinical outcomes in selected patients with advanced ovarian cancer [3,4,5,6]. Initially, the use of these agents was associated with improved clinical outcomes compared to placebo in patients with BRCA1/2 mutations [3,4]. Recent data demonstrated improved progression-free survival (PFS) in patients with advanced epithelial ovarian cancer after a complete or partial response to first-line platinum-based chemotherapy who received maintenance treatment with olaparib combined with bevacizumab compared to bevacizumab alone in patients with homologous recombination deficiency (HRD) [6]. In the Paola/ENGOT-ov25 phase III trial, the 5-year overall survival (OS) rates in patients with *BRCA1/2* mutations and/or with HRD-positive newly diagnosed advanced ovarian cancer were 65% and 55%, respectively, with olaparib and bevacizumab as a maintenance treatment [7]. Finally, maintenance treatment with niraparib was associated with improved PFS compared to placebo, irrespectively of HRD status [5]. However, clinical benefit was higher in patients with *BRCA1/2* mutations and/or HRD compared to patients with homologous recombination-proficient (HRP) tumors [5,6]. Finally, in the DUO-O trial, the use of maintenance therapy with bevacizumab in combination with durvalumab and olaparib in patients with newly diagnosed advanced ovarian cancer and no BRCA mutations resulted in a significantly greater PFS compared to bevacizumab monotherapy [8]. While the benefit from PARPi is significant in the first line, data from the trials in platinum-sensitive recurrent ovarian cancer do not show differences in OS for patients who received PARPi maintenance therapy compared to those who did not [9].

While *BRCA1/2* mutations are strong biomarkers for predicting benefit from PARPi in patients with epithelial ovarian cancer, they cannot accurately identify all patients who will benefit from these agents. Therefore, additional biomarkers are being investigated to increase the number of patients who will benefit from these treatments, while sparing the rest from unnecessary toxicity. HRD, which is also predictive of benefit from PARPi, characterizes 50% of ovarian high-grade serous tumors [10]. Importantly, HRD is associated not only with mutations in the homologous recombination repair (HRR) genes, but also with other molecular alterations, including *BRCA1* promoter methylation [11,12], *EMSY* amplification [10,13] and *PTEN* loss [10,14]. HRD can be detected using two molecular testing strategies. One strategy involves the investigation of the cause of HRD, whereas the second strategy involves the evaluation of tumor phenotype, by assessing genomic instability. Among available assays, the Myriad MyChoice^®^ CDx assay (Myriad Genetics, Salt Lake City, UT, USA) has been approved as a companion diagnostic for the use of PARPi [15]. Myriad MyChoice^®^ CDx assesses mutations in *BRCA1* and *BRCA2* along with genomic instability, by evaluating loss of heterozygosity (LOH), telomeric allelic imbalance and large-scale state transitions. The assay provides information on the presence of pathogenic mutations in *BRCA1/BRCA2*, along with a genomic instability (GI) score. The tumor is considered HRD-positive if a *BRCA1/2* mutation is identified and/or the GI score is ≥42. This assay has been used in large phase III clinical trials evaluating treatment with PARPi [5,6] and is the only test approved by the Food and Drug Administration (FDA). Despite the indisputable predictive value of this assay, several limitations complicate its use, including increased cost, the proportion of tumors with undetermined HRD status and the extended benefit of PARPi in HR-proficient tumors [5,6].

Additional in vitro diagnostic tests assessing HRD tumor status are being developed to improve patient selection for PARPi, including but not limited to the AmoyDx^®^ HRD Focus Panel (Amoy Diagnostics Co., Xiamen City, China), OncoScan™ (Thermo Fisher Scientific, Waltham, MA, USA), SOPHiA DDM HRD Solution (SOPHiA Genetics, Lausanne, Switzerland) and FoundationOne CDxTM (Foundation Medicine, San Diego, CA, USA). These platforms define HRD status by using diverse methods to assess genomic instability, performing complex bioinformatic analyses and using different positivity cut-offs. Specifically, the OncoScan™ CNV test utilizes single-nucleotide polymorphism genotyping and is generally acknowledged as a conventional method for conducting comprehensive genome-wide analysis of copy number alterations. Consequently, it can be employed for the identification of chromosomal regions with gain or loss and loss of heterozygosity (LOH), thereby providing a cost-effective alternative to commercially available next-generation sequencing (NGS) tests utilized for predicting HRD status [16,17]. In addition, the AmoyDx^®^ HRD Focus Panel uses NGS to qualitatively determine HRD status, via identifying and classifying single nucleotide variants and insertions and deletions in protein-coding regions and intron/exon boundaries of the BRCA1 and BRCA2 genes, thus determining a Genomic Scar Score [18]. Similarly, FoundationOne^®^ CDx detects, via NGS substitutions, insertion and deletion alterations and copy number alterations in 324 genes and select gene rearrangements, to qualitatively provide HRD status, among other molecular alterations [19]. Whether these assays can accurately identify specific groups of patients who will benefit from PARPi has yet to be determined.

In this study, formalin-fixed paraffin-embedded tumor tissue samples from 50 patients with newly diagnosed EOC and known Myriad MyChoice HRD status were centrally reassessed and re-evaluated for HRD status with two alternative platforms, the AmoyDx^®^ HRD Focus Panel and OncoScan™, blinded for the Myriad MyChoice CDx HRD status. Our aim was to evaluate the concordance in HRD status and/or GI status between Myriad MyChoice^®^ CDx assay and the two alternative platforms.

## 2. Materials and Methods

### 2.1. Patients

The present study comprised 50 formalin-fixed paraffin-embedded (FFPE) tumor tissue blocks, obtained from patients with epithelial ovarian cancer. Patients had been treated at Departments of Medical Oncology, affiliated with the Hellenic Cooperative Oncology Group (HeCOG). All patients had undergone tumor tissue profiling using the Myriad MyChoice^®^ CDx assay. HRD status, GI score and the presence of *BRCA1/2* mutations were recorded from Myriad’s reports. Patient clinicopathological characteristics and outcome data were recorded from patient medical records. The data collection was conducted in compliance with the regulations of the bioethics committees of the participating hospitals. The study was approved by the Institutional Review Board of General Hospital “Papanikolaou” (424/3.6.2022).

### 2.2. Tumor Tissue Processing and DNA Isolation

Tumor tissue processing and DNA extraction were performed at the Laboratory of Molecular Oncology (LMO), Hellenic Foundation for Cancer Research (HeFCR)/Aristotle University of Thessaloniki. Based on study protocol, tumor DNA was isolated from the FFPE tumor block, previously employed for HRD status assessment using the Myriad MyChoice CDx test. Alternatively, if tumor tissue was not adequate for further analysis, another FFPE block from the corresponding patient would be retrieved.

Available FFPE tumor blocks were subjected to histological review by an experienced pathologist to evaluate hematoxylin-eosin-stained sections for confirmation of diagnosis and tumor cell content (TCC%). Additionally, tumor dense areas were marked for manual macrodissection, prior to DNA extraction, in order to enrich samples for tumor DNA. TCC was assessed as an approximate metric for FFPE tumor DNA in the extracted samples, corresponding to tumor nuclei vs. all nuclei in the areas marked for macrodissection, as previously described [20]. FFPE tumor DNA extraction was then performed from 10 μm FFPE whole sections, following manual macrodissection, according to standard protocols with the QIAamp DNA Mini kit (Qiagen, Hilden, Germany). Once DNA concentration was measured with the Qubit fluorometer (Life Technologies, Paisley, UK), DNA aliquots were distributed in individual laboratories to carry out HRD status evaluation with the predefined platforms (AmoyDx^®^ HRD Focus Panel and OncoScan™). HRD status, as defined by Myriad MyChoice CDx assay, was blinded for the laboratories evaluating the alternative assays.

### 2.3. Targeted DNA NGS Analysis

In parallel, targeted DNA NGS analysis of selected tumors was performed using the validated Oncomine™ BRCA Research Assay (Thermo Fisher Scientific, Waltham, MA, USA) to screen the entire coding region of *BRCA1* and *BRCA2* genes for the presence of all classes of mutations, namely SNVs, indels and exon or whole gene deletion or duplication events (implemented at the LMO). Library preparation with the Oncomine BRCA Research Assay was performed with standard protocols, according to the manufacturer’s instructions (Life Technologies/Ion Torrent, Carlsbad, CA, USA). Resulting libraries were clonally amplified on the One-Touch-2 instrument, enriched on the OneTouch ES and sequenced on an Ion Proton sequencer. Data retrieval and base calling were performed on the Torrent Server (5.12.3). Consequently, the Oncomine BRCA Research Somatic-530-w3.6-DNA-Single Sample Ion Reporter Workflow (v5.18) was applied to automatically annotate identified variants.

### 2.4. AmoyDx^®^ HRD Focus Panel

DNA concentration was measured with the QUBIT DNA HS Assay kit (Invitrogen™, Waltham, MA, USA). Library preparation was performed using the AmoyDx^®^ HRD Focus Panel kit (Amoy Diagnostics Co., Xiamen City, China) according to the manufacturer’s instructions and sequenced using an Illumina High Output Kit on a NextSeq 500/550 platform. *BRCA1* and *BRCA2* variant information were obtained by an AmoyDx NGS data analysis system (ANDAS) (Amoy Diagnostics Co., Ltd.). SEQUENZA (v3.0.0) [21] was utilized with the default parameters to detect allele-specific CNVs, using the read depth and B-allele frequency of the SNPs with GC content correction to determine tumor allele-specific CNVs, tumor purity, and ploidy.

Using the segmentation data and allele-specific CNVs, the features of the Genomic Scar (GS) model [18] were prepared. Adjacent segments were merged if they had the same CNV status in both alleles. Chromosomal CN segments were then divided into three categories according to their physical properties, which included the length of CN (large: >15 Mb, middle: 10–15 Mb, small: 5–10 Mb), site of CN (telomere, centromere or other position), and type of CN (LOH, allele-specific CNV, allele-balanced CNV). A total of twenty-eight CNV features were constructed, consisting of three CNV lengths multiplied by three CNV sites multiplied by three CNV types, as well as the number of breakpoints across the genome. Then the CNV events of each sample were counted according to the kinds of CNV features present, and the GS scores were calculated using the GS model [18] with these features. Finally, the GI status (positive threshold greater than or equal to 50) was obtained.

### 2.5. OncoScan™ (OncoScan Copy Number Variations (CNVs) Assay)

#### 2.5.1. OncoScan Data Analysis

Hybridization was carried out on OncoScan™. The Chromosome Analysis Suite (ChAS) was used for the primary analysis of .CEL files and quality control calculations (MAPD, ndSNPQC). ASCAT (v3.0.0) (allele-specific copy number analysis of tumors) [22,23] using logR ratio and B-allele frequency of autosomal markers with GC content and replication timing correction was used to evaluate and calculate tumor purity, ploidy, and allele-specific copy number profiles.

#### 2.5.2. OncoScan GI Score Analysis Algorithm

Segmentation data from ASCAT, along with the previously described algorithms [24] and definitions, were used to calculate LOH [25], the number of telomeric-allelic imbalances (NTAIs) [26] and large-scale state transitions (LSTs) [27]. The NTAI score was calculated as the number of regions with allelic imbalance that extend to one of the subtelomeres, do not cross the centromere and are longer than 11 Mb [28]. The total value of genomic instability was the sum of the three components and was given as a score (GI score, positive threshold > 42 based on cut-off used by the Myriad MyChoice^®^ CDx assay).

The GI score bioinformatics algorithm (RediScore v1.0) incorporated modifications adjusted to the OncoScan array. The minimum number of probes/SNPs for a TAI region was adjusted to 126 fitting the OncoScan genome-wide resolution and chromosome-specific ploidy by major copy number fraction was determined. The most recent version of the GI score bioinformatics algorithm is further described in Tsantikidi et al., 2023 [17].

### 2.6. Statistical Analysis

Descriptive statistics (counts with percentages for categorical and median values with the corresponding ranges for continuous variables) were used to summarize patient and tumor characteristics and other variables of interest. The primary endpoint of the study was the assessment of concordance in HRD status between the approved Myriad MyChoice^®^ CDx assay and the two other platforms (AmoyDx^®^ HRD Focus Panel and OncoScan™). Concordance was assessed among the various methods in terms of (a) GI score as a continuous variable, (b) GI status as a categorical variable (positive–negative) based on GI score and (c) HRD status as a categorical variable (positive–negative) based on GI score and/or presence of *BRCA1/2* mutation. Pearson’s correlation coefficient was used to investigate the correlation between the GI score of the Myriad MyChoice^®^ CDx assay and the GI score from each of the other two platforms. Concerning the use of GI score from each of the two platforms as a predictor of the final HRD, receiver operating characteristic (ROC) curves were constructed. For each curve, AUC was calculated, along with the 95% confidence interval. As for the prediction of HRD status using categorical risk factors, 2 × 2 confusion matrices were derived, and sensitivity, specificity, positive and negative predicted values (PPV and NPV, respectively) were calculated, along with the 95% confidence intervals. Lastly, in order to select an optimal threshold value (cut-off point) for OncoScan™, the Youden index was utilized.

PFS was defined as the time interval from the initiation of first-line treatment to the date of discontinuation (due to any reason), disease progression and death from any cause or last contact, whichever occurred first. Best response during first-line treatment was defined per physician’s assessment locally at each medical department. Analysis was performed using R v.4.1.1.

## 3. Results

### 3.1. Patient Characteristics

Overall, this study included tumor tissue samples from 50 patients with a median age of 60 (range 38–84), diagnosed from 2/2019 to 5/2022. The majority of the patients had been diagnosed with high-grade serous epithelial ovarian cancer (45 patients, 90%). Overall, 24 (48%) patients with advanced disease received treatment with PARPi, as maintenance treatment, either as monotherapy or in combination with bevacizumab. The patients’ detailed clinicopathological characteristics are shown in Table 1.

#### 3.1.1. Tumor Tissue Evaluation

In all tumor samples used in the analysis, DNA extraction was performed from the FFPE block initially employed for HRD status assessment with the Myriad MyChoice CDx assay. No additional FFPE tissue sample was used for the analysis. Overall, the mean TCC% for the study’s tumor DNA samples was 63% (median 60%; range 10–100%; 75% with ≥50% TCC). Samples with TCC ≥ 10% were further analyzed, with 46 samples of them having TCC ≥30%. Finally, all resulting tumor DNA samples passed Qubit quantity control (DNA concentration ≥ 10 ng/μL). Samples were then processed for HRD status assessment with the aforementioned platforms, as well as targeted NGS sequencing with the Oncomine™ BRCA Research Assay. Duplicate samples using an alternative FFPE tumor tissue block from three patients were also evaluated (Supplementary Materials).

#### 3.1.2. Concordance among Different HRD Assays

Based on the Myriad MyChoice CDx assay, 28 of 50 patients (56%) had a positive HRD result and were characterized as HRD: 8/28 (28.6%) with a positive GI status with pathogenic *BRCA1/2* mutations, 15/28 (53.6%) with a positive GI status without pathogenic or likely pathogenic *BRCA1/BRCA2* mutations and 5/28 (17.8%) with a negative GI status or inconclusive results but pathogenic or likely pathogenic *BRCA1/BRCA2* mutations. In two (4%) patients, the assay yielded inconclusive results and no HRD status was assigned to these tumors. In 3 (of 28) additional patients, although no GI score was provided, the tumors were characterized as HRD (two harbored a *BRCA1/2* mutation and one did not have a specific explanation).

Overall, tumor samples from 50 patients were evaluated with the AmoyDx^®^ HRD Focus Panel and from 43 with OncoScan™. Four samples were excluded from the final analysis of the AmoyDx^®^ HRD Focus Panel due to low TCC (<30%), as per the manufacturer’s suggestion. Both platforms provided results for all tumors.

#### 3.1.3. Concordance in Terms of GI Score

**Figure 1:**
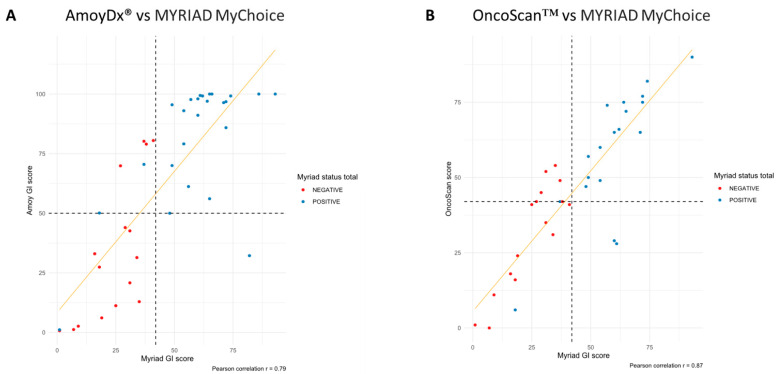
Correlation between Myriad MyChoice and each alternative homologous recombination deficiency (HRD) assay using genomic instability (GI) score. (**A**) Myriad MyChoice vs. AmoyDx^®^ HRD Focus Panel and (**B**) Myriad MyChoice vs. OncoScan™.

When evaluating the GI score as a continuous variable, correlation analysis showed that the AmoyDx^®^ HRD Focus Panel and OncoScan™ were highly correlated with Myriad MyChoice GI score (r = 0.79 and r = 0.87, respectively) (Table 2, Figure 1). In addition, the respective AUCs for the two methods were 0.897 (95% CI = 0.804–0.991) for the AmoyDx^®^ HRD Focus Panel and 0.853 (95% CI = 0.728–0.977) for OncoScan™, demonstrating that each method can be used to discriminate between samples classified as HRD-positive or-negative by Myriad MyChoice.

#### 3.1.4. Concordance in Terms of GI Status

**Figure 2:**
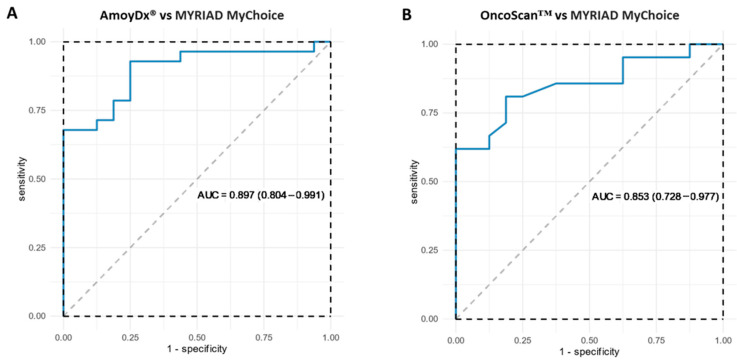
Areas under the curve for each HRD assay as predictor of Myriad MyChoice GI status: (**A**) AmoyDx^®^ HRD Focus Panel and (**B**) OncoScan™.

Concordance between each alternative method and Myriad MyChoice was assessed in terms of the GI status (positive–negative) and after excluding inconclusive samples. The sensitivity, specificity, PPV and NPV for each method is shown in Table 2. Based on the analysis, each method could accurately predict HRD-positive status in a high proportion of ovarian tumors, while specificity (predicting HRD-negative status) was lower for both methods. Areas under the curve (AUCs) for each HRD assay as a predictor of Myriad MyChoice GI status are demonstrated in Figure 2.

In all five tumors with inconclusive results in terms of GI score based on Myriad MyChoice, the two alternative methods, the AmoyDx^®^ HRD Focus Panel and OncoScan™, provided similar GI status (three positive and two negative). Details on discordant cases can be found in Table 3.

Finally, in order to select an optimal threshold value (cut-off point) for the OncoScan™, the Youden index was utilized [29]. The Youden cut-off of 47 was estimated based on the binormal ROC curve analysis. This value is highly similar to the predefined cut-off value, which confirms the high agreement level of the different GIS calculation pipelines [29,30]. Analysis of the concordance metrics using the cut-off of 47 is shown in Supplementary Table S1.

#### 3.1.5. Mutations in BRCA1/2

Among pathogenic *BRCA1* or *BRCA2* mutations, five involved Large Genomic Rearrangements (LGRs), encompassing one or more exons. Of these, three were on *BRCA1* and two on *BRCA2*, while at least four of them were clinically actionable genetic findings. The concordance of *BRCA1/2* status between the AmoyDx^®^ HRD Focus Panel and Myriad MyChoice are shown in Supplementary Table S2. The concordance between the Oncomine™ BRCA Research Assay and Myriad MyChoice was not assessed, due to the non-blinded status of the investigators evaluating this assay. The positive and negative predictive values of the AmoyDx^®^ HRD Focus Panel were 70% and 83.3%, respectively.

As described earlier, there were three tumors with *BRCA1/2* mutations that had low GI scores. While all tumors were predicted as positive by the AmoyDx^®^ HRD Focus Panel, one had a low GI score both with the AmoyDx^®^ HRD Focus Panel and OncoScan™. The remaining two had positive scores. Interestingly, these three patients received first-line platinum-based treatment, two followed by maintenance treatment, and all remain free of recurrence 2.5 years from treatment initiation.

#### 3.1.6. Concordance in Terms of Final HRD Status

Results based on the analysis of the HRD status (using both GI status and the presence of *BRCA1/2* pathogenic variants) are shown in Table 3. The sensitivity, specificity, PPV and NPV were only assessed for AmoyDx^®^ HRD Focus Panel/Myriad MyChoice (Table 3). Analysis was not performed for OncoScan™ because the assessment of *BRCA1/2* mutations which accompanies GI score calculation was performed in a different lab (Laboratory of Molecular Oncology), was not part of the same pipeline and might introduce additional bias.

#### 3.1.7. Mutations in HRR Genes

In addition to *BRCA1/2*, pathogenic variants were detected in other homologous recombination genes, including *ATM*, *BRIP1*, *CHEK2, PALB2*, *RAD51C* and *RAD51D*. One tumor, with a negative GI status by Myriad MyChoice, had four *ATM* loss-of-function variants and a pathogenic *BRCA2* variant. Another tumor that was GI-positive with Myriad MyChoice had both a splice-disrupting variant in *RAD51C* and a pathogenic *BRCA1* variant. Two tumors found GI-positive by Myriad MyChoice were devoid of pathogenic or likely pathogenic *BRCA1/BRCA2* variants, but one harbored a pathogenic *RAD51C* variant and the other two loss-of-function *PALB2* variants.

Contrarily, a pathogenic *CHEK2* variant was detected in a patient with a GI-negative tumor, while a patient’s tumor with an inconclusive GI status harbored a likely pathogenic *FANCL* variant and a splice variant in *ATM*. Both of these tumors were negative for pathogenic *BRCA1* and *BRCA1* variants.

#### 3.1.8. Patients’ Clinical Outcomes

The majority of the patients received first-line platinum-based treatment (39 patients, 78%); of these 14 (20%) received neoadjuvant treatment and underwent interval debulking, while the rest of the patients underwent surgery and chemotherapy subsequently. Among patients with advanced disease who received first-line treatment, 29 (74.4%) achieved partial or complete responses. Two-thirds of the patients (33 patients, 66%) received maintenance treatment with PARPi (9 patients, 27%), bevacizumab (9, 27%) or with combination therapy with olaparib and bevacizumab (15, 45%). Details in Table 1.

**Figure 3:**
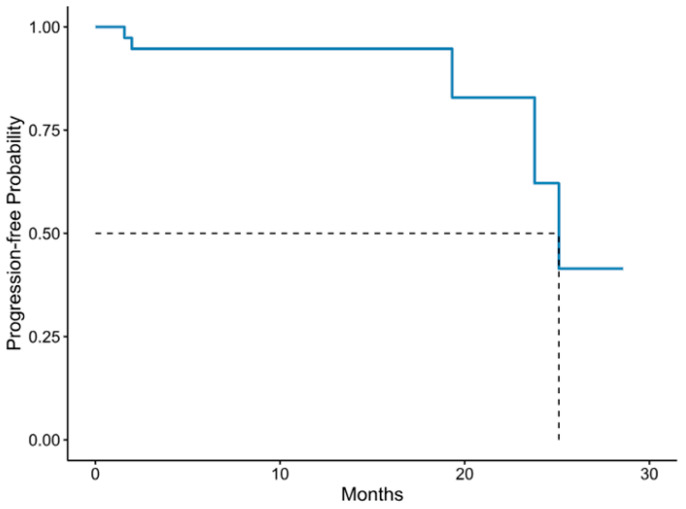
Progression-free survival (PFS) in patients with advanced-stage ovarian cancer. Solid blue line = PFS, Dashed black line = median PFS.

The median PFS of the patients of our study was 25.1 months (95% C.I: 0.6–12) (Figure 3). All three patients with a low Myriad MyChoice GI score but with *BRCA1/2* pathogenic variants had a complete response to first-line chemotherapy and had not progressed by the completion of the analysis. No association between HRD status and clinical outcomes was evaluated due to the small sample size and underpowered analysis.

## 4. Discussion

This is the first study to compare results between the standard-of-care Myriad MyChoice and two alternative assays evaluating HRD status in ovarian cancer, using a blinding methodology for the laboratories evaluating the assays. Importantly, all three assays were performed using DNA from the same tumor tissue sample, thus limiting discordance rates due to cancer heterogeneity. Our findings demonstrated high concordance among Myriad MyChoice assay and each assay under evaluation, including the AmoyDx^®^ HRD Focus Panel and OncoScan™. In detail, concordance was high both in terms of GI score (continuous) and status (categorical) and final HRD status, after taking into account GI score and *BRCA1/2* mutations. These assays, if further validated, can be used as robust alternative options to identify patients with epithelial ovarian cancer who will have a greater benefit from treatment with PARPi alone or in combination with bevacizumab.

Previous studies have compared different assays assessing HRD with the Myriad MyChoice assay. The AmoyDx^®^ HRD Focus Panel has been previously reported to have a high agreement with the Myriad MyChoice CDx assay (87.8%, in 65 of 74 tumors evaluated, HRD results were concordant). All nine discordant cases involved tumors that were predicted as HR-proficient by the Myriad MyChoice assay and were subsequently predicted as HRD by the AmoyDx assay [31]. Another study reported 100% concordance in HRD status between the AmoyDx^®^ HRD Focus Panel and Myriad MyChoice CDx assays, in 13 patients with epithelial ovarian cancer [32]. The investigators further evaluated the underlying mechanisms leading to HRD positivity, including mutations in HRR-related genes and copy numbers of PTEN and EMSY. Other preliminary results from studies comparing HRD status evaluated with the AmoyDx^®^ HRD Focus Panel, OncoScan™ and Myriad MyChoice CDx demonstrated substantial concordance between the three assays (k > 0.75 for all comparisons) [30]. Among tests based on commercially available SNP-based platforms, OncoScan™ has the highest correlation with the HRD score and status as determined by the Myriad MyChoice CDx assay [16].

Other methods evaluating HRD status, including Illumina’s TruSightTM Oncology 500 [33] and FoundationOne CDxTM [19], have also been compared to Myriad MyChoice CDx. After initial concordance calculations, optimization was performed to improve the agreement. The overall percentage agreement was 96% (92.2–97.9) [34]. Finally, the ENGOT European HRD initiative developed the “Leuven” HRD test on ovarian cancer tumor tissues from the biobank of University Hospitals Leuven, to provide and validate an alternative academic laboratory-developed HRD assay, comparable to the Myriad MyChoice^®^ CDx assay [35]. Using the “Leuven” HRD assay, the HRD status of 468 available ovarian tumor samples of patients who participated in the PAOLA-1/ENGOT-ov25 trial was determined. Overall percent agreement with the Myriad MyChoice^®^ CDx assay was 91%, with positive and negative percent agreement being 94% and 86%, respectively [35].

Our study demonstrated high sensitivity rates of the alternative methods evaluated. However, none of the methods demonstrated fully equivalent results to the Myriad MyChoice assay, mostly due to low specificity rates. Selected HRD-negative tumors as predicted by Myriad MyChoice assay were predicted to be HRD-positive by the other assays. While the Myriad MyChoice assay is the gold standard assay, questions arise as to whether alternative assays may detect additional HRD-positive tumors. In addition, as previously shown, the optimization of assays under evaluation and their cut-offs may improve accuracy in detecting HRD [30,34]. In our study, we investigated an optimized positivity cut-off point of the OncoScan™ assay, using the Youden index, to improve the classifier. However, the number of samples in this cohort may introduce overfitting biases, and, therefore, the results need to be interpreted with caution. Prospective external validation of the different methods and optimized cut-offs is warranted before their wide use in clinical practice.

Assessing HRD assays in diverse patient populations is critical, since the sensitivity and specificity of these assays may differ due to unique tumor and patient characteristics. For instance, LGRs may not be detected by currently used HRD assays, which is critical for selected patients [36,37,38]. LGRs involve types of genetic variants that alter significantly part of a gene, frequently encompassing one or more adjacent exons. LGRs can be a result of deletion, duplication or inversion and, therefore, can disrupt the sequence of a gene, resulting in a loss of function of tumor suppressor genes. Next-generation sequencing approaches are optimally designed to detect single nucleotide variants and small indels, with LGRs being frequently neglected. Identification and characterization of LGRs are essential in both germline and tumor testing, especially in populations with high incidence rates of these phenomena. In fact, in the Greek population, there have been identified four LGRs disrupting the BRCA1 C-terminal (BRCT) domain of *BRCA1* and one LGR deleting exons 12 and 13 of *BRCA2*, all of which have been shown to be founders. Among Greek patients with breast and ovarian cancer, *BRCA1* and *BRCA2* LGRs alone comprise up to 24% of pathogenic variants identified in *BRCA1* and *BRCA2* [39,40,41,42,43,44,45]. In our study, selected LGRs were not detected by the assays under evaluation. It is evident, therefore, that methods failing to detect LGRs will miss a considerable fraction of genetic events that will be impactful on prevention and treatment. Ways to overcome this limitation, especially in populations where these alterations are more commonly identified, are needed.

Despite the small patient population in our study, several questions arise for the use of HDR assays in daily clinical practice. First, in our study, we noted selected tumors with *BRCA1/2* mutations but with low GI scores, either assessed by Myriad MyChoice or an alternative HRD assay. Whether these patients derive equal benefit from PARPi to patients with high GI scores remains to be elucidated. Second, selected patients have tumors with HRD GI scores just below the cut-off used for HRD positivity. These patients may also benefit from PARPi. The utilization of the GI score as a continuous variable or additional methods to assess benefit from PARPi might merit higher predictive value. Third, analysis of selected tumor tissue samples derived inconclusive results despite appropriate quality control metrics of the respective samples. It is critical to determine the optimal management for patients with inconclusive results. Another question that arises is whether the number of patients for whom a physician’s clinical decision regarding the use of PARPi would be altered based on the different HRD result (i.e., HRD vs. not HRD or HRD vs. indeterminant) is significant. Due to the small sample size and our trial design, it was difficult to conduct such an analysis and, therefore, larger trials are needed to shed some light into this matter. Finally, in our study, we demonstrated that analyses of different tumor tissue samples from the same patient provided certain discordant results in terms of GI status. Selecting the most appropriate tissue sample is of great clinical significance, as it sets the base for patient treatment selection.

While a significant proportion of patients will benefit from PARPi, the majority will eventually recur. Diverse mechanisms of resistance have been implicated in resistance to treatment with PARPi [46,47]. Treatment strategies to overcome resistance, including combinations of PARPi with antiangiogenic agents, PI3K/AKT pathway inhibitors, epigenetic drugs and ATR/CHK1 inhibitors are being evaluated [46,47].

Limitations of our study include the retrospective nature and the relatively small number of tumor tissue samples. In addition, a different number of samples was evaluated by each alternative assay, further limiting the head-to-head comparison of these assays. Finally, despite the presence of fully annotated clinical data, our study was not powered to address correlations with clinicopathological and outcome data. Strengths of our study include the evaluation of two alternative assays assessing HRD status, the use of the same tumor tissue block for all three assays, the use of the same DNA aliquot for the two alternative assays and the single-blinded design of the analysis, with the different labs evaluating the alternative assays not being aware of the initial HRD status and GI score provided by the Myriad MyChoice assay.

## 5. Conclusions

In conclusion, our study demonstrated high concordance between the Myriad MyChoice assay and each assay under evaluation, including the AmoyDx^®^ HRD Focus Panel and OncoScan™. Having multiple available assays to identify patients with HRD-positive tumors is critical to ensure sufficient diagnostic capacity across different countries, as well as to provide greater variety for clinicians and diagnostic laboratories. Factors that need to be taken into account when selecting a method to assess HRD status include the sensitivity and specificity of the method, cost and reimbursement potential depending on the country, turnaround time, and the availability of local molecular laboratory testing. Importantly, prospective independent validation of each assay is critical to ensure the accurate selection of patients who will benefit from PARPi, while sparing the rest from unnecessary treatment and toxicity.

## Figures and Tables

**Table 1:** Patient characteristics.

Characteristic	Total *n* = 50
**Age at diagnosis**	
**Median (min, max)**	60 (38–84)
**Histological subtype (*n* = 50)**	***n* (%)**
Serous	45 (90.0)
Clear cell	3 (6.0)
Endometrioid	2 (4.0)
**Family history cancer (*n* = 47)**	
No	33 (70.2)
Yes	14 (29.8)
**Family history breast/ovarian cancer (*n* = 47)**	
No	42 (89.4)
Yes	5 (10.6)
**Performance status (*n* = 45)**	
0	36 (80)
1	6 (13.3)
2	3 (6.7)
3	0
4	0
**Stage at diagnosis**	
I	3
II	7
III	30
IV	10
**Neoadjuvant treatment (*n* = 50)**	
Yes	14 (20.0)
No	36 (80.0)
**First-line treatment (*n* = 50)**	
Yes	39 (78.0)
No	11 (22.0)
**Maintenance treatment (*n* = 50)**	
Yes	33 (66.0)
No	17 (34.0)
**Response to first-line treatment (*n* = 39)**	
Complete response/no evidence of disease	17 (43.6)
Partial response	12 (30.8)
Stable disease	7 (17.9)
Disease progression	3 (7.7)
**Maintenance treatment (*n* = 33)**	
PARP inhibitor	9 (27.3)
Bevacizumab	9 (27.3)
PARP inhibitor/bevacizumab	15 (45.4)

Abbreviations: *n*: number, PARP: poly(ADP-ribose)-polymerase.

**Table 2:** Concordance characteristics of Myriad MyChoice and the two additional assays in terms of homologous recombination deficiency (HRD) status based on either genomic instability (GI) status or HRD (GI status and/or BRCA1/2 mutations) status, after excluding the inconclusive/failed Myriad MyChoice samples. Analysis for HRD status was not performed for OncoScan™ since the assessment of *BRCA1/2* mutations which accompanies GI score calculation was performed in a different lab.

				Myriad MyChoice^®^ CDx						
	Metric	*n*	Result	+	−	OPA	Sensitivity	Specificity	PPV	NPV
**AmoyDx^®^ HRD Focus**	GI status	42	+	22	6	83.3% (68.6–93.3%)	95.7% (78.1–99.9)	68.4% (43.4–87.4)	78.6%	92.9%
			−	1	13					
	HRD	44	+	28	5	88.6% (75.4–96.2)	100% (87.7–100)	68.8% (41.3–89)	84.8%	100%
			−	0	11					
**OncoScan™**	GI status	40	+	16	7	77.5% (61.5–89.2%)	88.9% (65.3–98.6)	68.1% (45.1–86.1)	69.6%	88.2%
			−	2	15					

Abbreviations: HRD: homologous recombination deficiency, GI: genomic instability, OPA: overall percentage agreement, PPV: positive predictive value, NPV: negative predictive value.

**Table 3:** Discordant cases between Myriad MyChoice and AmoyDx and OncoScan™.

	HRD		GI Status			BRCA1/2 Mutations	
ID	Myriad	Amoy	Myriad	Amoy	OncoScan	Myriad	Amoy
1	Positive	Positive	Positive	Positive	Positive	No	No
2	Positive	Positive	Positive	Negative	-	Yes	Yes
3	Negative	Negative	Negative	Negative	Negative	No	No
4	Negative	Negative	Negative	Negative	Negative	No	No
5	Positive	Positive	Positive	Positive	Positive	Yes	Yes
6	Positive	Positive	Positive	Positive	Negative	No	No
7	Negative	Negative	Negative	Negative	Positive	No	No
8	Positive	Positive	Positive	Positive	Positive	No	No
9	Positive	Positive	Positive	Positive	Positive	Yes	Yes
10	Negative	Negative	Negative	Negative	Negative	No	No
11	Negative	Negative *	Negative	Negative *	Negative	No	No
12	Positive	Positive	Positive	Positive	Positive	No	No
13	Positive	Positive	Positive	Positive	Negative	No	No
14	Negative	Negative	Negative	Negative	Positive	No	No
15	Positive	Positive	Positive	Positive	Positive	No	No
16	Positive	Positive	Negative	Positive	Positive	Yes	Yes
17	Inconclusive	Negative	Inconclusive	Negative	Negative	No	No
18	Negative	Positive	Negative	Negative	Negative	No	Yes
19	Positive	Positive	Positive	Positive	Positive	No	No
20	Positive	Positive	Positive	Positive	Positive	Yes	No
21	Positive	Positive	Positive	Positive	Positive	No	Yes
22	Negative	Negative	Negative	Negative	Positive	No	No
23	Positive	Positive	Positive	Positive	Positive	No	No
24	Positive	Positive	Positive	Positive	-	Yes	No
25	Positive	Positive	Positive	Positive	-	Yes	No
26	Positive	Positive	Positive	Positive	Positive	No	No
27	Positive	Positive	Positive	Positive	Positive	No	Yes
28	Positive	Positive	Positive	Positive	Positive	No	No
29	Inconclusive	Negative	Inconclusive	Negative	Negative	No	No
30	Negative	Negative *	Negative	Negative *	Negative	No	No
31	Negative	Negative *	Negative	Negative *	Negative	No	No
32	Positive	Positive	Positive	Positive	Positive	No	No
33	Negative	Negative	Negative	Negative	Negative	No	No
34	Negative	Negative	Negative	Negative	Negative	No	No
35	Negative	Positive	Negative	Positive	Negative	No	No
36	Positive	Positive	Positive	Positive	-	Yes	No
37	Negative	Positive	Negative	Positive	Positive	No	No
38	Positive	Positive	Positive	Positive	-	Yes	Yes
39	Positive	Positive	Negative	Negative	-	Yes	Yes
40	Negative	Negative *	Negative	Negative *	Negative	No	No
41	Positive	Positive	Inconclusive	Positive	-	Yes	Yes
42	Positive	Positive	Positive	Positive	Positive	No	No
43	Negative	Positive	Negative	Positive	Positive	No	No
44	Positive	Positive	Inconclusive	Positive	Positive	Yes	No
45	Negative	Negative	Negative	Negative	Negative	No	No
46	Positive	Positive	Negative	Positive	Negative	Yes	No
47	Negative	Negative	Negative	Negative	Negative	No	No
48	Negative	Positive	Negative	Positive	Positive	No	No
49	Negative	Negative	Negative	Negative	Negative	No	No
50	Positive	Positive	Positive	Positive	Positive	No	No

* Excluded from the analyses due to low TCC.

## Data Availability

The data underlying this article are available in the article and in its online Supplementary Materials.

## Supplementary Materials

The following supporting information can be downloaded at: https://www.mdpi.com/article/10.3390/cancers15235525/s1, Table S1: Cut-off optimization; Table S2: Concordance metrics on Myriad MyChoice and AmoyDx^®^ HRD Focus evaluating *BRCA1/2* mutations in terms of *BRCA1/2* status as a categorical variable (*BRCA1/2*+: P/LP variants identified–*BRCA1/2*−: no variants identified).
