# Supplementary material for: Concordance between Three Homologous Recombination Deficiency (HRD) Assays in Patients with High-Grade Epithelial Ovarian Cancer
Source: Cancers (Basel). 2023 Nov 22;15(23):5525. doi: 10.3390/cancers15235525 (PMC10705222; doi:10.3390/cancers15235525)
Supplement: Supplementary file 1 [file cancers-15-05525-s001.zip › cancers-2660840-supplementary.pdf]

Supplement

Cut-off optimization

In order to select an optimal threshold value (cut-off point) for the OncoScan™, the Youden Index was utilized. The Youden cut-off of 47 was estimated based on the binormal ROC curve analysis. Analysis of the concordance metrics using the cut-off of 47 is included below.

Supplemental Table S1. Cut-off optimization.

			Myriad myChoice® CDx GI status					
**Assay ***	**N**	Result	**+**	**-**	**Sensitivity**	**Specificity**	**PPV**	**NPV**
**OncoScan™ GI status (cut-off 47)**	40	**+**	16	3	88.9% (65.3-98.6)	86.4% (65.1-97.1)	84.2%	90.5%
		**-**	2	19				

* Concordance with the 2rd method was not assessed, due to the non-blinded status of the investigators evaluating this assay.

Supplemental Table S2: Concordance metrics on Myriad MyChoice and AmoyDx® HRD Focus evaluating *BRCA1/2* mutations in terms of *BRCA1/2* status as a categorical variable (*BRCA1/2*+: P/LP variants identified–*BRCA1/2*−: no variants identified).

			Myriad myChoice® CDx BRCA1/2 status					
**Assay ***	**N**	Result	**+**	**−**	**Sensitivity**	**Specificity**	**PPV**	**NPV**
**AmoyDx® HRD Focus BRCA1/2 status**	46	+	7	3	53.8% (25.1-80.8)	90.9% (75.7-98.1)	70%	83.3%
		−	6	30				

Abbreviations: HRD: Homologous Recombination Deficiency, PPV: Positive Predictive Value, NPV: Negative Predictive Value

* Concordance with the 2rd method was not assessed, due to the non-blinded status of the investigators evaluating this assay.

Duplicate samples

In addition to 50 samples evaluated for HRD status using alternative assays, we evaluated three duplicate samples using an alternative FFPE tumor tissue block. Although analysis could not be performed due to the small sample size, we observed that HRD status was predicted similarly with AmoyDx® using the different tumor blocks. However, GI Status was different in one pair (GI status 32.2 - negative vs 77.3 - positive) with AmoyDx®; the final HRD status remained the same in both cases, due to the presence of a *BRCA1* mutation in those tumors. Duplicate samples were not evaluated with the OncoScan™ assay.
